# Non-Small Cell Lung Cancer Presenting as a Mobile Left Atrial Mass

**DOI:** 10.7759/cureus.27859

**Published:** 2022-08-10

**Authors:** Ali H Allouch, Hassan A Zaeiter, Rachad M Harb, Louay I Dandach

**Affiliations:** 1 Oncology, Lebanese University, Faculty of Medical Sciences, Beirut, LBN; 2 Cardiology, Lebanese University, Faculty of Medical Sciences, Beirut, LBN

**Keywords:** cardiac oncology, diastolic murmur, cardiac tumors, left atrial mass, non small cell lung cancer

## Abstract

Primary cardiac tumors are extremely rare and mostly metastatic in origin. The signs and symptoms depend on the location of the tumor rather than its histopathology and, rarely, may be the first presentation of the malignant disease. We report a 54-year-old woman diagnosed with non-small lung cancer with new-onset heart murmur and dyspnea on exertion as the first clinical manifestations.

## Introduction

Most cardiac tumors are metastatic in origin rather than primary and originate from other primary malignancies, mainly lung cancer, esophageal cancer, melanoma, lymphoma, and sarcoma. These primary tumors can spread to the heart through the hematogenous, lymphatic system or even by direct extension and may involve any cardiac structure, most commonly the pericardium and less commonly the endocardium [1-2].

Cardiac involvement in lung cancer represents 8% to 10 % of cases [3], and the invasion of the left atrium (LA) is a rare entity but well-documented in the literature [4-5].

In a previous review, the incidence of pulmonary vein involvement accounts for 4.2% of cases of primary lung cancer while the involvement of pulmonary veins with extension into the left atrium was reported in 0.9% of cases as in our case [3]. Rarely, the primary malignancy is diagnosed due to the signs and symptoms of cardiac involvement [6-7].

Herein, we present a patient diagnosed with non-small cell lung cancer (NSCLC) due to cardiac involvement as the first manifestation of the disease. This case serves as a reminder that lung cancer may present with uncommon features like a cardiac tumor, and physicians must do an extensive workup when faced with a cardiac mass.

## Case presentation

A 54-year-old female patient, a previous smoker, presented for new-onset fatigue and dyspnea on mild exertion, no chest pain, occasional cough without hemoptysis, and no weight loss. Her past medical history was significant for an old ischemic cerebrovascular accident (CVA) with residual distal right-handed weakness.

On physical examination, she was tachycardic with normal blood pressure and oxygen saturation in room air, cardiac auscultation revealed a low-pitched, grade II/VI rumbling diastolic murmur suggested mitral stenosis, and pulmonary auscultation revealed mild crackles in the lower third of the left hemithorax.

**Figure 1:**
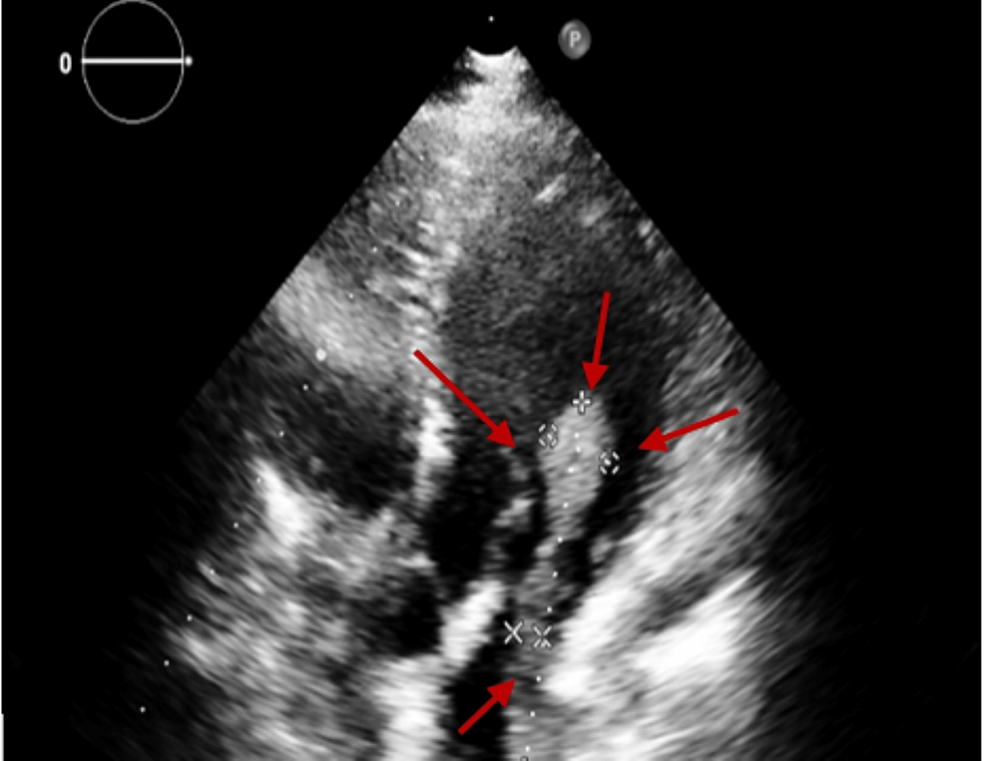
Transthoracic echocardiography modified apical five-chamber view showing an elongated mass (1.6 cm x 0.6 cm x 6.3 cm) protruding into the left ventricle during diastole

**Figure 2:**
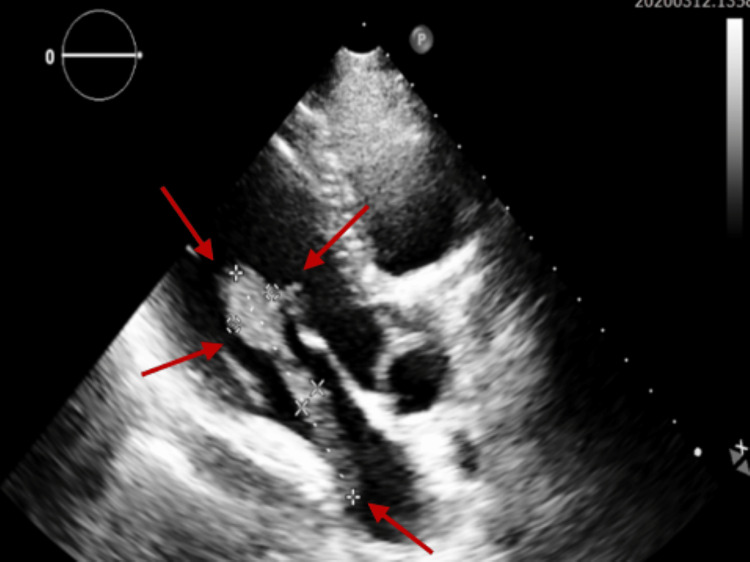
Transthoracic echocardiography modified long-axis view, showing an elongated mass (1.6 cm x 0.6 cm x 6.3 cm) protruding into the left ventricle during diastole

Transthoracic echocardiography (Figure 1, Figure 2) was done and showed a large, elongated mass in the left atrium of 6.9 cm x 1.4 cm protruding into the left ventricle in diastole without significant effect on the mitral flow, which is probably a direct extension from a left lung mass through a pulmonary vein, normal left atrium volume, and left ventricular (LV) function.

**Figure 3:**
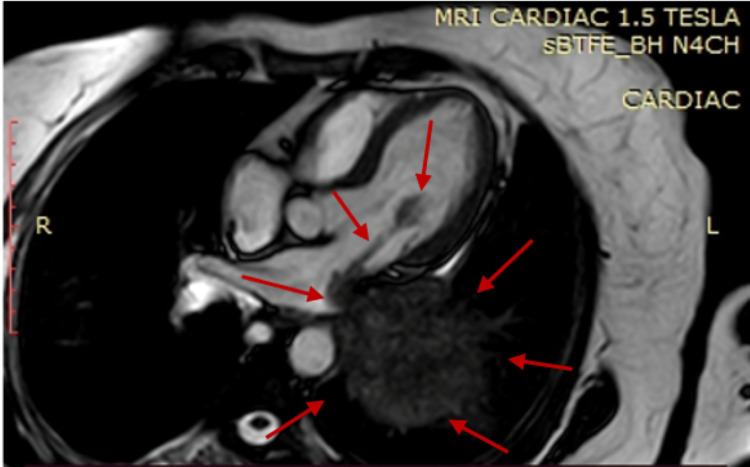
Cardiac MRI showing the left lung lower lobe mass (11 cm x 11 cm x 8 cm) entering the left atrium through the pulmonary vein, elongated left atrial mass (5.6 cm x 1 cm) inside the left atrium

**Figure 4:**
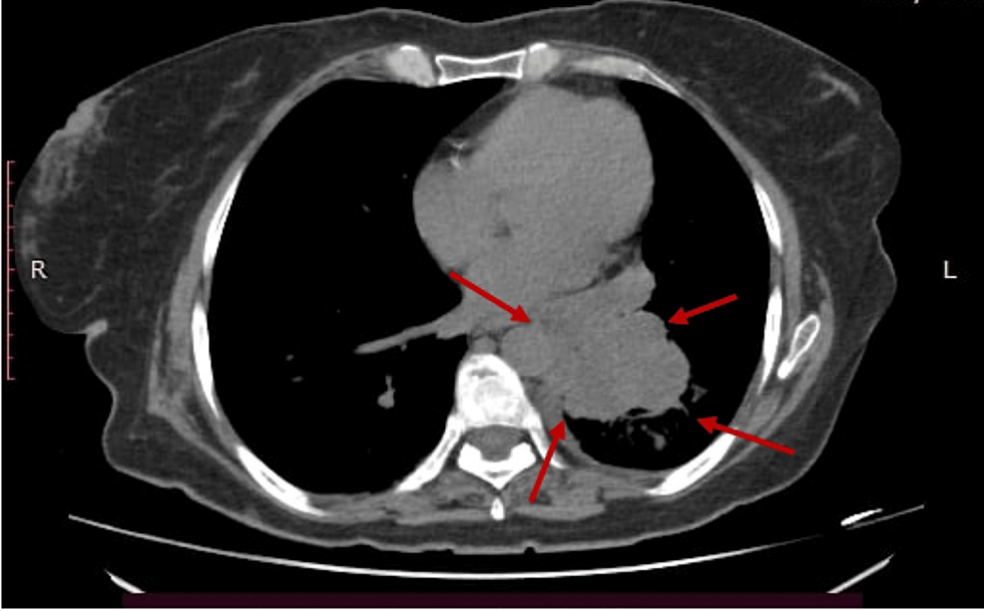
CT scan showing the left lower lobe lung mass

The finding was confirmed by cardiac and chest MRI (Figure 3) that showed a large (11 cm x 11 cm x 8 cm) left lung lower lobe mass entering the left atrium (LA) through the pulmonary vein, with possibly a direct invasion of the lateral atrial wall to form an elongated mass (5.6 cm x 1 cm) inside the left atrium, very highly mobile and enters through the mitral valve into the left ventricle during each cardiac cycle. Also, a CT scan was done, and it showed the left lower lobe mass in the lung (Figure 4).

The patient then underwent bronchoscopy with tissue biopsy that found a malignant cell consisting of NSCLC. The patient's situation deteriorated rapidly before surgical treatment, and she died from a massive ischemic CVA and cardiopulmonary arrest in the ICU ward.

## Discussion

Left atrial invasion by lung carcinoma through a pulmonary vein was reported in a few cases in the literature [8]. Qianli et al. reported a patient with squamous cell carcinoma invading the left atrium through pulmonary veins, with no lymph node involvement, who underwent neo-adjuvant chemotherapy followed by surgical resection with cardiopulmonary bypass of the primary tumor and then followed by adjuvant chemotherapy without any post-operative complications. The patient was found to have solitary brain metastasis seven months after surgery [3].

Lestuzzi et al. from Italy reported a rare case of small-cell lung carcinoma extending through the pulmonary veins and invading the left atrium [9]. Desai et al. reported a 46-year-old female patient who was diagnosed with squamous cell carcinoma spreading through the right superior pulmonary veins into the left atrium as seen on echocardiography and confirmed by trans-esophageal echocardiography [10]. Watanabe and Kubo reported a similar case with the same histology [8].

Lung cancer with cardiac invasion can not only be asymptomatic but can also cause and present with serious and life-threatening complications that require urgent medical or invasive intervention including arrhythmias, heart failure, atrioventricular nodal block, myocardial infarction requiring urgent angiography, pericardial effusion and tamponade requiring urgent pericardiocentesis or pericardial window or even mechanical obstruction of circulation or distal embolization to the brain and systemic circulation [11-12].

Concerning the treatment approach, lung cancer with cardiac invasion is considered a locally advanced disease (T4 in the TNM classification), and, in most cases, it is considered unresectable. However, lately, some T4 tumors are considered for surgical resection with neo-adjuvant and/or adjuvant chemotherapy [12-13]. The surgical option is based on the risk versus benefit of the surgical and non-surgical approaches and a multidisciplinary tumor approach is necessary to take such a serious decision [5]. Taking into consideration that surgical resection using a cardiopulmonary bypass of the primary lung cancer with left atrial resection can improve the quality of life and translate to an improvement in performance status, a decrease in symptoms severity, and decreased risk of death due to the cardiac complications or tumor emboli [12-13].

In the case of metastatic disease, patients with good performance status are eligible for systemic chemotherapy or, in the new era of lung cancer, for targeted therapy and immunotherapy [14-15]. Also, radiotherapy is a modality that can be used in patients with brain metastasis and includes stereotactic radiosurgery, which is used in oligometastatic disease, and whole brain radiotherapy, which is used less often and indicated in patients with more than three metastases [16-18].

## Conclusions

Lung cancer is considered the leading cause of cancer deaths worldwide in both men and women. Cardiac involvement accounts for 10% and is rarely diagnosed due to its signs and symptoms. However. when present, it is considered a locally advanced disease and should be treated rapidly due to serious health-threatening complications.
